# Performance of Three Commercial Rapid Diagnostic Tests for Detection of IgM and IgG Antibodies Against SARS‐CoV‐2

**DOI:** 10.1002/iid3.70441

**Published:** 2026-04-26

**Authors:** Moyra Machado Portilho, Stephane Fraga de Oliveira Tosta, Maysa Pellizzaro, Rosângela Oliveira dos Anjos, Elaine Carvalho de Oliveira, Pamela dos Santos Nascimento de Santana, Patrícia Sousa dos Santos Moreira, Leile Camila Jacob‐Nascimento, Mirela Maisa da Silva Souza, Deborah Bittencourt Mothé, Cláudia Brodskyn, Mitermayer Galvão Reis, Cristiane Wanderley Cardoso, Guilherme Sousa Ribeiro

**Affiliations:** ^1^ Instituto Gonçalo Moniz Fundação Oswaldo Cruz Salvador Brazil; ^2^ Secretaria Municipal de Saúde de Salvador Salvador Brazil; ^3^ Escola de Medicina Veterinária e Zootecnia Universidade Federal da Bahia Salvador Brazil; ^4^ Faculdade de Medicina Universidade Federal da Bahia Salvador Brazil; ^5^ Yale School of Public Health New Haven Connecticut USA

**Keywords:** COVID‐19, IgG, IgM, Rapid test, SARS‐COV‐2, serological test

## Abstract

**Background:**

Despite widespread vaccination, SARS‐CoV‐2 transmission continues, and serological testing remains relevant for selected diagnostic scenarios and population‐based assessments of antibody responses. Rapid diagnostic tests (RDTs) for SARS‐CoV‐2 antibodies are attractive for field use and decentralized settings, but their diagnostic performance varies and requires independent evaluation. We assessed the performance of three commercially available lateral flow RDTs (PANBIO™ COVID‐19 IgG/IgM Rapid Test Device, Bio‐Manguinhos‐Fiocruz TR COVID‐19 (IgM‐IgG), and Bio‐Manguinhos‐Fiocruz TR DPP® COVID‐19 IgM/IgG) in Salvador, Brazil.

**Methods:**

Using blind analyses, we evaluated 257 serum samples from RT‐PCR‐confirmed cases and 199 control samples from individuals with other febrile illnesses or healthy donors collected before and during the pandemic.

**Results:**

Overall sensitivity for IgM or IgG detection was limited across all tests (52%–58%), while specificity was high for two assays (97%–98%) and lower for one (80%). Sensitivity peaked between 11 and 20 days after symptom onset (80%–91%) and declined thereafter. Among 75 vaccinated individuals without prior COVID‐19, antibody positivity ranged from 39% to 55%.

**Conclusion:**

These findings indicate that the evaluated RDTs had high specificity but insufficient sensitivity for reliable clinical diagnosis or for assessing vaccination status in serological surveys. Our results support cautious use of these assays and highlight the need for more accurate and robust antibody‐based rapid tests to strengthen immunological surveillance and public health preparedness in the post‐pandemic period.

## 1 Introduction

COVID‐19 emerged as a major public health emergency in recent history. As of September 2025, the global death toll from COVID‐19 has reached 7.1 million [1]. The introduction of vaccines was a game‐changer, significantly reducing the number of severe cases [2] and deaths [3]. However, the ongoing emergence of SARS‐CoV‐2 (severe acute respiratory syndrome coronavirus 2) variants poses a risk, as some may evade immunity from prior infection or vaccination. This underscores the need to monitor transmission and assess population levels of anti‐SARS‐CoV‐2 antibodies, which serve as proxies for infection and vaccine coverage. Therefore, accurate methods for detecting infection remain essential.

Diagnosis of COVID‐19 has predominantly relied on direct methods, such as real‐time reverse transcription polymerase chain reaction (RT‐PCR) and antigen detection using immunodiagnostic techniques, commonly referred to as rapid diagnostic tests (Ag‐RDTs), performed on nasal or nasopharyngeal samples [4]. Serological tests to detect antibodies against SARS‐CoV‐2 were widely employed at the onset of the pandemic [5]. However, their use declined as access to direct tests expanded, as these are more sensitive in the early stages after symptom onset and can differentiate between a current infection and a prior infection or vaccination. Nevertheless, antibody assays remain relevant in specific diagnostic scenarios, particularly when patients present beyond the optimal window for direct viral detection. In addition, serological testing continues to play an important role in population‐based serosurveys, allowing the assessment of cumulative infection, hybrid immunity, and antibody persistence following vaccination or natural infection [6, 7].

Rapid diagnostic tests (RDTs) for detecting IgM and IgG antibodies against SARS‐CoV‐2 can be valuable in these contexts because they are non‐invasive, easy to perform, and provide results in approximately 15 min, making them suitable for point‐of‐care or field use [8, 9]. These characteristics are particularly relevant in low‐resource settings, during large‐scale serosurveys, and in decentralized health systems where access to laboratory‐based serological assays remains limited. However, not all commercially available rapid diagnostic tests have been extensively evaluated by independent teams, despite their continued availability and use in surveillance activities and public procurement programs. In this context, independent evaluations of commercially available serological RDTs remain essential to inform their appropriate use in post‐pandemic surveillance, clinical decision‐making in selected scenarios, and public health preparedness.

Here, we describe a comparative investigation of the performance of three lateral flow RDTs for detecting IgM and IgG antibodies against SARS‐CoV‐2: 1) the PANBIO™ COVID‐19 IgG/IgM Rapid Test Device (Abbott, Jena, Germany), 2) the Bio‐Manguinhos TR COVID‐19 (IgM‐IgG) (Bio‐Manguinhos, Rio de Janeiro, Brazil), and 3) the Bio‐Manguinhos TR DPP® COVID‐19 IgM/IgG (Bio‐Manguinhos, Rio de Janeiro, Brazil). These RDTs were selected for evaluation because they are approved by the Brazilian Health Regulatory Agency (ANVISA) and are commercially available in Brazil.

## 2 Materials & Methods

### 2.1 Study Design and Sample Selection

**Figure 1:**
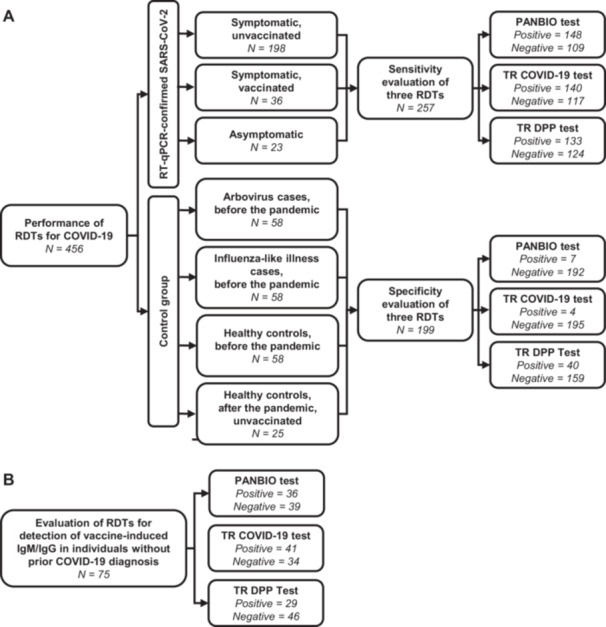
Flowchart of participants included in the evaluation of three rapid diagnostic tests (RDTs). Panel A shows participants assessed for RDT performance in detecting IgM and IgG antibodies against SARS‐CoV‐2. Panel B shows participants included in the assessment of RDTs for detection of vaccine‐induced IgM and IgG antibodies.

This study was reported according to the Standards for Reporting of Diagnostic Accuracy (STARD) Guideline (Supplementary File 1). To evaluate the sensitivity and specificity of the RDTs, we used a panel of sera from 456 individuals (one sample per individual) from Salvador, a large urban center in Brazil (population: 2.4 million) (Figure 1A). The sensitivity panel included 257 sera specifically collected for this study between December 8, 2020, and May 12, 2021, from individuals who tested positive for SARS‐CoV‐2 by RT‐PCR in nasopharyngeal samples. Of these, 36 were COVID‐19 patients with prior vaccination, 198 were unvaccinated COVID‐19 patients, and 23 were asymptomatic individuals who underwent RT‐PCR testing because they lived with a symptomatic individual who had RT‐PCR‐confirmed COVID‐19 (5 vaccinated, 18 unvaccinated). This panel of 257 samples was sufficient to estimate a sensitivity of at least 88% with 95% confidence and a precision of ±4%, based on standard binomial methods for estimating proportions using an adapted version of Cochran's formula.

Specificity was evaluated in 199 deidentified serum samples obtained from participants enrolled in other research study protocols. Of these, 174 were collected before the COVID‐19 pandemic from 58 patients with an influenza‐like illness (fever and respiratory symptoms), 58 patients with RT‐PCR‐confirmed arboviral infections (29 with dengue, 29 with chikungunya), and 58 healthy individuals. The samples from the patients with an influenza‐like illness, dengue, and chikungunya were collected during the convalescent phase of the disease (between 10 and 47 days after the onset of symptoms) between October 21, 2016, and December 18, 2019, as part of a long‐term acute febrile illness (AFI) enhanced surveillance study aimed at detecting arboviral infections in a public emergency health unit in Salvador. Details of the AFI surveillance protocol have been previously described [10, 11, 12]. The 58 serum samples from healthy individuals were collected between September 10 and November 25, 2019, as part of a community‐based cohort study aimed at investigating the transmission dynamics of arbovirus infections. An additional 25 healthy individuals provided serum samples after the second wave of the COVID‐19 pandemic in Salvador (between March 1 and April 23, 2021). However, they reported no prior COVID‐19 diagnosis, respiratory symptoms during the COVID‐19 pandemic, or receipt of a COVID‐19 vaccination. This panel of 199 samples provided 95% confidence to estimate specificities of at least 91% with a precision of ±4%, using the same binomial proportion–based approach described above.

To determine whether the tests could detect antibodies elicited by vaccination against COVID‐19, we also included in the study 75 serum samples from healthy individuals who had been vaccinated but denied a confirmed diagnosis of COVID‐19 by RT‐PCR (Figure 1B). These deidentified sera were also obtained from participants enrolled in other research study protocols between August 18 and September 3, 2021.

All participants with an RT‐PCR‐confirmed diagnosis of COVID‐19, whose serum was collected for this study, were 18 years of age or older and provided written informed consent for their inclusion. The deidentified sera used as control to assess the tests' specificity and their ability to detect antibodies induced by COVID‐19 vaccination were obtained from participants who had provided written informed consent in the studies in which they were originally enrolled. For participants under 18 years of age, written assent was obtained in conjunction with written consent from their parents or guardians. The Brazilian National Research Ethics Committee (CONEP) approved this study, including the anonymous use of serum samples from participants of other studies (CAAE: 30609820.2.0000.0040). All adopted procedures are in accordance with the ethical standards of the Declaration of Helsinki.

### 2.2 Sample Testing

As previously mentioned, the RDTs evaluated were: 1) the PANBIO™ COVID‐19 IgG/IgM Rapid Test Device (Abbott, Jena, Germany; kit lot COV0042035), hereinafter referred to as the PANBIO test; 2) the Bio‐Manguinhos TR COVID‐19 IgM/IgG (Bio‐Manguinhos, Rio de Janeiro, Brazil; kit lot 20PDV001Z), hereinafter referred to as the TR COVID‐19 test; and 3) the Bio‐Manguinhos TR DPP® COVID‐19 IgM/IgG (Bio‐Manguinhos, Rio de Janeiro, Brazil; kit lot 207VD002Z), hereinafter referred to as the TR DPP test. The three RDTs were designed to detect both IgM and IgG antibodies against SARS‐CoV‐2. The PANBIO and TR DPP tests target the SARS‐CoV‐2 nucleocapsid protein, while the TR COVID‐19 test uses a combination of unspecified antigens. The procedures used during the evaluation of each test were in accordance with the manufacturer's instructions and are briefly outlined in Table 1 below.

**Table 1:** Characteristicvvated according to the manufacturer's instructions.

Test	Fabricant	Protein target	Sample volume	Buffer volume	Time until interpretation
PANBIO test^a^	Abbott	Nucleocapsid	10 µL	60 µL (2 drops)	10–20 min
TR COVID‐19 test^b^	Bio‐Manguinhos	Unspecified antigen combination	10 µL	70 µL (2 drops)	15 min
TR DPP test^c^	Bio‐Manguinhos	Nucleocapsid	10 µL (diluted in 150 µL of buffer)	270 µL (9 drops)	10–15 min

^a^ PANBIO™ COVID‐19 IgG/IgM Rapid Test Device.

^b^ TR COVID‐19 IgM/IgG.

^c^ TR DPP® COVID‐19 IgM/IgG.

All blood samples were refrigerated before centrifugation for serum separation, and the obtained sera were stored at −20°C until analysis. For the PANBIO test, 10 μL of serum and two drops (approximately 60 μL) of the test buffer were applied to the sample well. The results were visually interpreted after 10 to 20 min. For the TR COVID‐19 test, 10 μL of serum and 70 μL of test buffer were added to the device well, and the results were visually read after 15 min. To minimize bias in the interpretation of these two tests, all serum samples were assigned a secondary identification number before testing, and independent researchers performed blinded readings. In cases of discrepancy, a third researcher reviewed the test cassettes to determine the final result.

For the TR DPP test, 10 µL of serum was mixed with 150 µL of buffer, and 100 μL of the serum‐buffer mixture was added to the first well of the cassette. After 5 min, nine drops (approximately 270 µL) of buffer were added to the second well. Results were read after 10 to 15 min using the provided electronic microreader, which recorded the intensity of the IgM and IgG signals. A single researcher documented these readings. All samples were tested once, except in cases where invalid results required retesting.

### 2.3 Data Analysis

Absolute and relative frequencies, as well as medians and interquartile ranges (IQRs), were used to describe the demographic and clinical characteristics of individuals with RT‐PCR‐confirmed SARS‐CoV‐2 infection whose samples were used to assess the sensitivity of the RDTs.

Sensitivities with 95% confidence intervals were calculated separately for the IgM and IgG components and for the combined result (where a positive reaction in either component was considered positive). It was assessed for all individuals with RT‐PCR‐confirmed SARS‐CoV‐2 infection and stratified by symptom presence, prior COVID‐19 vaccination status, and the time between symptom onset and blood collection for symptomatic, unvaccinated individuals. This analysis was not conducted for symptomatic, vaccinated individuals due to the small sample size in this group.

Specificities with 95% confidence intervals were calculated separately for the IgM and IgG components and for the combined result (where negativity was defined as a negative reaction in both the IgM and IgG components). It was assessed overall and stratified by the origin of the control samples: convalescent cases of influenza‐like illness detected before the pandemic; convalescent cases of arbovirus disease – dengue or chikungunya – detected before the pandemic; healthy individuals with samples collected before the pandemic; and healthy individuals with samples collected after the pandemic who reported no COVID‐19 vaccination or diagnosis of COVID‐19.

Finally, we assessed the frequency and corresponding 95% confidence intervals with which the evaluated tests detected IgM, IgG, or at least one of these antibody classes in healthy individuals who had been vaccinated against COVID‐19 but had not reported receiving a confirmed diagnosis of the disease.

## 3 Results

The demographic and clinical characteristics of individuals with RT‐PCR‐confirmed SARS‐CoV‐2 infection, whose samples were used to assess the sensitivity of the RDTs, are described in Table 2. Clinical manifestations were typical of mild COVID‐19 and similar between vaccinated and unvaccinated individuals. Receiving medical care was reported by 63.3% of symptomatic unvaccinated individuals and 75.0% of symptomatic vaccinated individuals. Hospitalization and intensive care unit support were uncommon for both groups (< 6% and < 3%, respectively). None of the symptomatic cases received mechanical ventilation. The median time from symptom onset to blood sample collection was approximately 60 days for both vaccinated and unvaccinated symptomatic individuals, which should be considered when interpreting sensitivity estimates across time strata.

**Table 2:** Clinical characteristics of individuals with RT‐PCR‐confirmed SARS‐CoV‐2 infection included in the evaluation study of three rapid serological tests for COVID‐19.

	*n* (%), unless otherwise indicated		
Characteristics	RT‐PCR‐confirmed cases of SARS‐CoV‐2 infection		
	Symptomatic and not vaccinated (*N* = 198)	Symptomatic and vaccinated (*N* = 36)	Asymptomatic (*n* = 23)
Demographic			
Female sex	102 (51.5)	24 (66.7)	6 (26.1)
Median age in years (interquartile range)	40 (33.0–52.0)^a^	49.0 (41.0–61.0)	45.0 (40.0–53.5)
Time between the onset of symptoms and blood sample collection			
Median (interquartile range)	57.5 (24.3–131.8)	63.5 (44.0–95.0)	NA
Minimum‐maximum	0–230	18–217	NA
Reported signs and symptoms			
Headache	123 (62.1)	28 (77.8)	NA
Anosmia	108 (54.5)	17 (47.2)	NA
Fatigue	105 (53.0)	25 (69.4)	NA
Dysgeusia	92 (46.4)	16 (44.4)	NA
Myalgia	85 (42.9)	18 (50.0)	NA
Rhinorrhea	78 (39.4)	11 (30.6)	NA
Fever	65 (32.8)	17 (47.2)	NA
Diarrhea	64 (32.3)	14 (38.9)	NA
Sore throat	60 (30.3)	11 (30.6)	NA
Chills	57 (28.8)	15 (41.7)	NA
Dyspnea	47 (23.7)	10 (27.8)	NA
Nausea	40 (20.2)	10 (27.8)	NA
Cough	35 (17.8)	8 (22.2)	NA
Abdominal pain	30 (15.2)	8 (22.2)	NA
Redness in the eyes	29 (14.6)	9 (25.0)	NA
Vomit	16 (8.1)	4 (11.1)	NA
Rash	10 (5.1)	1 (2.8)	NA
Back pain	10 (5.1)	3 (8.3)	NA
Health care^b^			
Received health care	95 (63.3)	27 (75.0)	NA
Hospitalization	9 (6.0)	2 (5.5)	NA
ICU support	4 (2.7)	1 (2.8)	NA
Oxygen support	4 (2.7)	2 (5.5)	NA
Mechanical ventilation	0 (0.0)	0 (0.0)	NA
At least one dose of the COVID‐19 vaccine	0 (0.0)	36 (100.0)	5 (21.7)

Abbreviations: ICU = intensive care unit, NA = Not applicable.

^a^ It was not possible to retrieve the age information for one participant.

^b^ Healthcare data were available for 150 of the 198 participants symptomatic and not vaccinated.

The overall sensitivity for detecting IgM or IgG antibodies was 57.6% for the PANBIO test, 54.5% for the TR COVID‐19 test, and 51.8% for the TR DPP test (Table 3; Figure 1A). The corresponding 95% confidence intervals are shown in Supplementary Table 1. The sensitivity of the tests among symptomatic, unvaccinated individuals was similar to the overall evaluation (58.1%, 54.6%, and 51.5%, respectively). In contrast, higher sensitivity was observed in symptomatic, vaccinated individuals (75.0%, 75.0%, and 63.9%, respectively), while lower sensitivity was found among individuals with asymptomatic infections (26.1%, 21.7%, and 34.8%, respectively). Notably, the IgM component of the PANBIO and the TR DPP tests demonstrated lower sensitivity in detecting SARS‐CoV‐2 infection compared with the IgG component, whereas the IgM and IgG components of the TR‐COVID‐19 test performed similarly (Table 3).

**Table 3:** Sensitivity and specificity of three commercially available rapid diagnostic tests for detection of IgM and IgG antibodies against SARS‐CoV‐2.

		**Number of positive results (% sensitivity)**								
Accuracy by group	No. of samples	PANBIO™ COVID‐19 IgG/IgM Rapid Test Device			Bio‐Manguinhos TR COVID‐19 (IgM‐IgG)			Bio‐Manguinhos TR DPP® COVID‐19 IgM/IgG		
Sensitivity		IgM	IgG	IgM or IgG	IgM	IgG	IgM or IgG	IgM	IgG	IgM or IgG
**RT‐PCR‐confirmed SARS‐CoV‐2 infection, overall**	257	17 (6.6)	144 (56.0)	148 (57.6)	131 (51.0)	137 (53.3)	140 (54.5)	61 (23.7)	122 (47.5)	133 (51.8)
Symptomatic, unvaccinated	198	14 (7.1)	112 (56.6)	115 (58.1)	101 (51.0)	106 (53.5)	108 (54.6)	44 (22.2)	93 (47.0)	102 (51.5)
Symptomatic, vaccinated^a^	36	2 (5.6)	27 (75.0)	27 (75.0)	27 (75.0)	27 (75.0)	27 (75.0)	14 (38.9)	23 (63.9)	23 (63.9)
Asymptomatic	23	1 (4.3)	5 (21.7)	6 (26.1)	3 (13.0)	4 (17.4)	5 (21.7)	3 (13.0)	6 (26.1)	8 (34.8)

		**Number of negative results (% specificity)**								
**Specificity**		**IgM**	**IgG**	**IgM and IgG**	**IgM**	**IgG**	**IgM and IgG**	**IgM**	**IgG**	**IgM and IgG**
**Non‐SARS‐CoV‐2‐infected or vaccinated individuals, overall**	199	193 (97.0)	197 (99.0)	192 (96.5)	195 (98.0)	199 (100.0)	195 (98.0)	167 (83.9)	190 (95.5)	159 (79.9)
Convalescent influenza‐like illness cases	58	57 (98.3)	58 (100.0)	57 (98.3)	57 (98.3)	58 (100.0)	57 (98.3)	51 (87.9)	57 (98.3)	50 (86.2)
Convalescent arbovirus illness cases^b^	58	56 (96.6)	57 (98.3)	55 (94.8)	56 (96.6)	58 (100.0)	56 (96.6)	42 (72.4)	55 (94.8)	40 (69.0)
Healthy controls										
Before the pandemic	58	55 (94.8)	57 (98.3)	55 (94.8)	57 (98.3)	58 (100.0)	57 (98.3)	49 (84.5)	54 (93.1)	45 (77.6)
During the pandemic, unvaccinated^c^	25	25 (100.0)	25 (100.0)	25 (100.0)	25 (100.0)	25 (100.0)	25 (100.0)	25 (100.0)	24 (96.0)	24 (96.0)

^a^ The vaccinated RT‐PCR‐confirmed COVID‐19 cases have used at least one dose of the COVID‐19 vaccine produced by AstraZeneca/Oxford or CoronaVac/Butantan.

^b^ The convalescent arbovirus illness cases include 29 RT‐PCR‐confirmed dengue cases and 29 RT‐PCR‐confirmed chikungunya cases, for which serum samples were obtained during convalescence (11–50 days post‐onset of fever) in the pre‐pandemic period.

^c^ All the non‐vaccinated healthy controls included after the start of the COVID‐19 pandemic denied having a diagnosis of COVID‐19.

**Figure 2:**
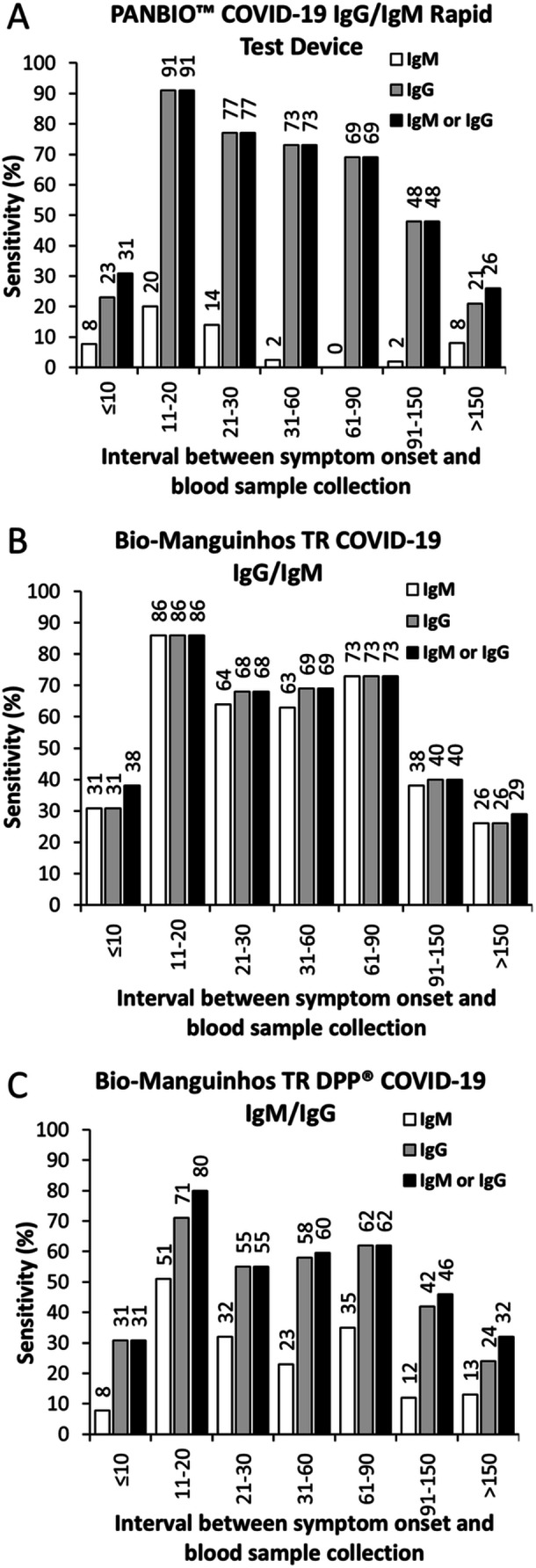
Sensitivity of three commercially available rapid diagnostic tests for detecting IgM and IgG against SARS‐CoV‐2, according to the interval between symptom onset and blood sample collection. Colors indicate the types of antibodies detected: IgM (white), IgG (gray), and IgM or IgG (black). (A) Sensitivity for the PANBIO™ COVID‐19 IgG/IgM Rapid Test Device. (B) Sensitivity for the Bio‐Manguinhos TR COVID‐19 IgM/IgG. (C) Sensitivity for the Bio‐Manguinhos TR DPP^®^ COVID‐19 IgM/IgG.

The sensitivity of the RDTs among symptomatic, unvaccinated individuals was also assessed based on the time between symptom onset and blood sample collection (Figure 2). For all three tests, sensitivity for detecting either IgM or IgG was low (< 40%) in samples collected within the first 10 days after symptom onset. It peaked between 11 and 20 days post‐symptom onset (91.0% for the PANBIO test, 86.0% for the TR COVID‐19 test, and 80.0% for the TR DPP test). Then, it gradually decreased, reaching its lowest levels in samples collected more than 150 days after the onset of symptoms. This pattern was consistent for the IgM and IgG components of all tests. Notably, across all intervals between symptom onset and sample collection, the IgG component of both the PANBIO and TR DPP tests demonstrated higher sensitivity than their respective IgM components. In contrast, the TR COVID‐19 test exhibited similar sensitivity for its IgM and IgG components over time.

The overall specificity, determined by negative results for both IgM and IgG antibodies, was 96.5% for the PANBIO test, 98.0% for the TR COVID‐19 test, and 79.9% for the TR DPP test (Table 3; Figure 1A). The corresponding 95% confidence intervals are provided in Supplementary Table 1. The PANBIO test and the TR COVID‐19 test demonstrated specificities ranging from 94.8% to 100% across all control sample groups evaluated, which included pre‐pandemic convalescent cases of influenza‐like illness and arbovirus illness (dengue or chikungunya), healthy individuals with pre‐pandemic samples, and healthy individuals with post‐pandemic samples who denied COVID‐19 vaccination or a diagnosis of COVID‐19. A similar pattern was observed for these two tests when considering only the IgM or IgG components. In contrast, the TR DPP test demonstrated high specificity (96.0%) for samples collected from healthy individuals who denied both COVID‐19 vaccination and disease after the pandemic began, but lower specificity for the other groups (ranging from 69.0% to 86.2%). This lower performance was primarily attributed to the IgM component of the test, which exhibited lower specificity compared to the IgG component, except for samples from healthy individuals who denied both COVID‐19 vaccination and diagnosis.

When evaluating the capacity of tests to detect IgM or IgG antibodies elicited by COVID‐19 vaccination, we used samples from healthy individuals who had not reported prior COVID‐19 infection but had received at least one dose of a COVID‐19 vaccine. Overall positivity rates were 48.0% for the PANBIO test, 54.7% for the TR COVID‐19 test, and 38.7% for the TR DPP test (Table 4; Figure 1B). The corresponding 95% confidence intervals are presented in Supplementary Table 2. The IgM components of the PANBIO and TR DPP tests showed lower positivity (5.3% and 12.0%, respectively) compared to their IgG components (44.0% and 29.3%, respectively) (Table 3). Conversely, for the TR COVID‐19 test, the IgM positivity (52.0%) was similar to the IgG positivity (48.0%).

**Table 4:** Positivity rates for IgM and IgG antibody detection among COVID‐19 vaccinated individuals using three commercial rapid diagnostic tests for SARS‐CoV‐2.

		Number of positive results (positivity rate in %)								
Detection of antibodies among vaccinated healthy individuals^a^	No. of samples	PANBIO™ COVID‐19 IgG/IgM Rapid Test Device			Bio‐Manguinhos TR COVID‐19 (IgM‐IgG)			Bio‐Manguinhos TR DPP® COVID‐19 IgM/IgG		
Group		IgM	IgG	IgM or IgG	IgM	IgG	IgM or IgG	IgM	IgG	IgM or IgG
Overall	75	4 (5.3)	33 (44.0)	36 (48.0)	39 (52.0)	36 (48.0)	41 (54.7)	9 (12.0)	22 (29.3)	29 (38.7)
By vaccine type										
CoronaVac (Sinovac/Butantan)	24	1 (4.2)	16 (66.7)	16 (66.7)	14 (58.3)	15 (62.5)	15 (62.5)	2 (8.3)	9 (37.5)	10 (41.7)
AstraZeneca/Oxford (Fiocruz)	26	1 (3.8)	9 (34.6)	10 (38.5)	13 (50.0)	12 (46.2)	14 (53.8)	4 (15.4)	8 (30.8)	11 (42.3)
Pfizer (BioNTech)	25	2 (8.0)	8 (32.0)	10 (40.0)	12 (48.0)	9 (36.0)	12 (48.0)	3 (12.0)	5 (20.0)	8 (32.0)
By the number of vaccine doses										
1 dose	34	1 (2.9)	15 (44.1)	16 (47.1)	18 (52.9)	17 (50.0)	19 (55.9)	3 (8.8)	11 (32.4)	13 (38.2)
2 doses	41	3 (7.3)	18 (43.9)	20 (48.8)	21 (51.2)	19 (46.3)	22 (53.7)	6 (14.6)	11 (26.8)	16 (39.0)
By time since last vaccine dose^b^^,^ c										
< 30 days	35	2 (5.7)	14 (40.0)	16 (45.7)	17 (48.6)	15 (42.9)	18 (51.4)	4 (11.4)	9 (25.7)	13 (37.1)
30‐89 days	16	2 (12.5)	6 (37.5)	7 (43.8)	8 (50.0)	7 (43.8)	8 (50.0)	2 (12.5)	4 (25.0)	5 (31.3)
≥ 90 days	23	0 (0.0)	12 (52.2)	12 (52.2)	13 (56.5)	12 (56.5)	14 (60.9)	2 (8.7)	9 (39.1)	10 (43.5)

^a^ After the start of the COVID‐19 pandemic, reported no priVaccinated healthy individuals were enrolled or diagnosis of COVID‐19, and had received at least one dose of one of the following vaccines: CoronaVac (Sinovac/Butantan), AstraZeneca/Oxford (Fiocruz), or Pfizer/BioNTech.

^b^ Time between the last vaccine dose and sample collection.

^c^ It was not possible to retrieve the vaccination date for one patient.

We further evaluated whether the positivity rate for detecting IgM or IgG antibodies differed by the type of COVID‐19 vaccine received (Table 4). Among samples from individuals vaccinated with CoronaVac, positivity rates were higher for the PANBIO and TR COVID‐19 tests (66.7% and 62.5%, respectively) than for the TR DPP test (41.7%). In contrast, samples from individuals vaccinated with AstraZeneca showed higher positivity for TR COVID‐19 (53.8%) than for PANBIO (38.5%) and the TR DPP (42.3%). Among individuals vaccinated with Pfizer, none of the tests reached a 50% positivity rate. We also assessed whether the number of vaccine doses affected antibody detection and found the tests exhibited similar positivity rates for IgM or IgG antibody detection in individuals who received one or two doses (Table 4).

Finally, we assessed whether the interval between the last vaccine dose and blood sample collection influenced positivity rates for IgM or IgG detection (Table 4). Each test showed similar positivity rates for samples obtained before 30 days and between 30 and 89 days post‐vaccination, with higher positivity observed in samples collected 90 days or more after vaccination. However, positivity rates for this latter group remained relatively low (52.2% for PANBIO, 60.9.% for TR COVID‐19, and 43.5% for TR DPP).

## 4 Discussion

We found that the PANBIO, TR COVID‐19, and TR DPP tests exhibited relatively low sensitivities (ranging from 51.8% to 57.6%) for detecting IgM or IgG antibodies against SARS‐CoV‐2 in a diverse set of individuals with RT‐PCR‐confirmed SARS‐CoV‐2 infection, which included symptomatic, unvaccinated individuals, symptomatic, vaccinated individuals, and asymptomatic individuals. The highest sensitivities were observed for the PANBIO and the TR COVID‐19 tests when used to detect antibodies in symptomatic and vaccinated individuals (75.0% for both). Although lower than the sensitivity observed for these two tests, the TR DPP test also showed higher sensitivity in the same group of samples (63.9%). In contrast, specificity was excellent for the PANBIO and TR COVID‐19 tests (> 95%), but moderate for the TR DPP (80% overall).

We found no prior reports on the accuracy of the TR COVID‐19 test. However, previous studies have evaluated the TR DPP test, with reported sensitivities ranging from 19.0% to 52.6% for IgM, 37.0% to 63.0% for IgG, and 68.8% when considering either IgM or IgG [13, 14], which is consistent with our findings. In contrast, studies assessing the sensitivity of the PANBIO test have produced conflicting results. Some investigations with RT‐PCR‐confirmed COVID‐19 patients reported sensitivities of 91.5% [15] and 95.2% [16] for samples collected more than 14 days after symptom onset, primarily driven by the IgG component. Conversely, a study evaluating two cohorts of RT‐PCR‐confirmed COVID‐19 patients from Spain reported lower sensitivities of 59.2% and 74.4% [17].

A likely explanation for the differences in sensitivity among these studies is the variation in the time elapsed between symptom onset and sample collection. Studies reporting high sensitivity for the PANBIO test had most samples collected between 14 and 56 days post‐symptom onset [15, 16]. In contrast, the study reporting lower sensitivity included patients earlier in the disease course, with a median of 7 days after symptom onset [17]. In our study, sensitivity was highest for all three tests when samples were obtained 11 to 20 days after symptom onset; for the PANBIO test, sensitivity reached 91%, consistent with previous findings for this time window. These results align with other studies evaluating RDTs, which found that sensitivity varies substantially depending on the timing of sample collection [13, 18] and the patient's clinical presentation. This underscores the importance of considering time since symptom onset when determining whether to use a COVID‐19 RDT.

Regarding the specificity of the three tests across a diverse panel of samples, the PANBIO and TR COVID‐19 tests exhibited specificities greater than 95% across all sample groups and test components. However, the TR DPP test showed lower specificities for the IgM component across all groups, except among non‐vaccinated healthy individuals during the pandemic period. This reduced the overall combined specificity. Similar specificities have been reported for the PANBIO test, with 98.7% specificity for IgG and 100% for IgM [14], and an overall specificity of 81.0% for the TR DPP test [13]. Conversely, Bernardes et al. reported higher specificities for the TR DPP test in symptomatic individuals, with 98% for IgM and 80% for IgG [14]. Differences in assay design and antigen presentation across RDTs may contribute to variability in background reactivity and, consequently, to differences in specificity estimates. However, the present study was not designed to identify the mechanistic basis of false‐positive results, and the lower specificity observed for the TR DPP test should therefore be interpreted as an empirical performance characteristic observed under the study conditions.

The mRNA (Pfizer/BioNTech) and viral vector (AstraZeneca/Oxford) vaccines elicit antibody production directed against the S protein [19, 20, 21]. However, the CoronaVac vaccine, developed by Sinovac Biotech Ltd. and widely used in Brazil early in the pandemic, is an inactivated vaccine that may induce a broader immune response. Of the three tests evaluated, PANBIO and TR DPP specifically target the nucleocapsid (N) protein, while TR COVID‐19 uses an unspecified combination of antigens. Therefore, RDTs targeting spike antibodies are more likely to detect vaccine‐induced responses for the first two vaccines and, likely, also for the CoronaVac vaccine, whereas RDTs targeting nucleocapsid antibodies would primarily reflect natural infection or vaccination with the whole‐virus CoronaVac vaccine. This likely explains the better performance of all RDTs evaluated in vaccinated individuals who received the CoronaVac vaccine and the higher sensitivity observed among RT‐PCR‐confirmed symptomatic vaccinated individuals compared to their non‐vaccinated counterparts.

Additionally, among healthy individuals with no history of COVID‐19 but who had received at least one vaccine dose, the proportion testing positive for IgM or IgG ranged from 38.7% to 54.7%. These findings suggest that, although the tests can detect vaccine‐induced antibodies, they are suboptimal for assessing prior vaccination status and should be used cautiously for this purpose. Furthermore, despite the high specificity of the PANBIO and the TR COVID‐19 tests in samples collected before the pandemic or from unvaccinated individuals during the pandemic, widespread vaccination complicates the interpretation of positive results. In this context, it may be challenging to determine whether a positive test result reflects an active or recent infection or is solely due to prior vaccination, thereby limiting the utility of these tests. Identifying vaccination‐related factors that influence positive test results could enhance the assessment of prior vaccination status. However, our subgroup analyses indicate that none of the tests achieve high positivity rates, regardless of vaccine type, number of doses, or time elapsed since the last dose.

In the current post‐vaccination context, these findings have broader implications for the continued use and development of SARS‐CoV‐2 serological tools. Assays developed and validated early in the pandemic were primarily intended for use in immunologically naïve populations and may no longer be fit‐for‐purpose in settings with high vaccine coverage and heterogeneous immune histories. In such contexts, qualitative rapid tests provide limited information for individual‐level diagnosis and are poorly suited for population‐based surveys aimed at distinguishing prior infection from vaccine‐induced immunity. These results underscore the need for next‐generation serological approaches that incorporate clearly defined antigen targets, quantitative or multiplex readouts, and validation frameworks explicitly designed for vaccinated populations and hybrid immunity.

Our study has some limitations. The sensitivity analysis was based on convenience samples collected in a single city between December 2020 and April 2021; therefore, we did not evaluate the diagnostic performance in samples from individuals infected with variants of concern that emerged later in the pandemic. Variants circulating during this period largely reflect the viral strains present at the time of assay development and initial deployment. In addition, the long median interval between symptom onset and sample collection may have influenced antibody levels, and the relatively small sample sizes in subgroup analyses may limit statistical power and generalizability. Finally, although we have included convalescent samples from individuals with influenza‐like illness, we did not assess specificity in samples obtained from patients with confirmed infections caused by other *Orthocoronavirinae* viruses, such as NL63, HCoV‐229E, MERS, or SARS‐CoV‐1, and prior infection with other human coronaviruses in both the influenza‐like illness, the confirmed arbovirus infection and the healthy control groups cannot be excluded. However, the high specificity of two assays (97%–98%) and the moderate specificity of one (80%) suggest that, if present, cross‐reactivity was unlikely to have substantially influenced our findings.

In conclusion, our study found that the PANBIO, TR COVID‐19, and TR DPP tests had overall sensitivities below 60% for detecting IgM or IgG in individuals with RT‐PCR‐confirmed SARS‐CoV‐2 infection, except among symptomatic vaccinated individuals, in whom sensitivity reached moderate levels of 75% for the PANBIO and TR COVID‐19 tests. The PANBIO and TR COVID‐19 tests demonstrated higher sensitivity for detecting IgM or IgG in samples collected between 11 and 20 days after symptom onset (91% and 86%, respectively). They also showed excellent specificities (> 96%) in samples collected before the pandemic or from unvaccinated individuals during the pandemic. However, interpreting positive results is challenging in the post‐vaccination context, because approximately 50% of vaccinated individuals who reported no history of SARS‐CoV‐2 infection tested positive. This indicates that a positive result may reflect either an acute or recent infection or antibodies induced by vaccination. Additionally, the tests are not reliable for detecting prior vaccination alone. Therefore, we conclude that these early‐generation qualitative RDTs have limited utility, as they cannot reliably distinguish between prior infection and vaccination or confirm either status with sufficient accuracy, restricting their use for individual diagnosis or serosurveillance. These findings underscore the need for serological tools specifically designed and validated for populations with widespread vaccination and hybrid immunity.

## Author Contributions

**Moyra Machado Portilho:** investigation, methodology, formal analysis, writing – original draft, writing – review and editing, conceptualization. **Stephane Fraga de Oliveira Tosta:** formal analysis, visualization, writing – original draft, writing – review and editing, methodology, investigation, funding acquisition. **Maysa Pellizzaro:** investigation; writing – review and editing. **Rosângela Oliveira dos Anjos:** investigation, writing – review and editing. **Elaine Carvalho de Oliveira:** investigation, writing – review and editing. **Pamela dos Santos Nascimento de Santana:** investigation, writing – review and editing. **Patrícia Sousa dos Santos Moreira:** investigation, writing – review and editing. **Leile Camila Jacob‐Nascimento:** investigation, writing – review and editing. **Mirela Maisa da Silva Souza:** investigation, writing – review and editing. **Deborah Bittencourt Mothé:** investigation, writing – review and editing. **Cláudia Brodskyn:** writing – review and editing, resources, methodology, funding acquisition, resources, methodology, funding acquisition. **Mitermayer Galvão Reis:** writing – review and editing, resources. **Cristiane Wanderley Cardoso:** investigation, methodology, writing – review & editing, conceptualization. **Guilherme Sousa Ribeiro:** investigation, methodology, writing – original draft, writing – review and editing, conceptualization.

## Statement Regarding the use of Artificial Intelligence

During the preparation of this manuscript, the authors used Grammarly and ChatGPT to assist with grammar and stylistic improvements. The authors subsequently reviewed and edited the content and take full responsibility for the publication.

## Ethics Statement

The Brazilian National Research Ethics Committee (CONEP) approved this study, including the anonymous use of serum samples from participants of other studies (CAAE: 30609820.2.0000.0040).

## Conflicts of Interest

The authors declare no conflicts of interest.

## Supporting information

Supporting File 1

Supporting File 2

Supporting File 3

## Data Availability

The data that support the findings of this study are available upon reasonable request to the corresponding author.

## References

[1] WHO . WHO Coronavirus (COVID‐19) Dashboard [Internet]. 2024 [cited 2025 September 3]. Available from: https://covid19.who.int.

[2] C. Graña , L. Ghosn , T. Evrenoglou , et al. Efficacy and safety of COVID‐19 vaccines ‐ Graña, C ‐ 2022 | Cochrane Library, [cited 2024 Oct 21], https://www.cochranelibrary.com/cdsr/doi/10.1002/14651858.CD015477/full. 10.1002/14651858.CD015477 PMC9726273 36473651

[3] L. Nab , E. P. K. Parker , C. D. Andrews , et al., “Changes in COVID‐19‐related Mortality Across Key Demographic and Clinical Subgroups in England From 2020 to 2022: A Retrospective Cohort Study Using the OpenSAFELY Platform,” Lancet Public Health 8, no. 5 (2023): e364–e377. 37120260 10.1016/S2468-2667(23)00079-8 PMC10139026

[4] Y. Mardian , H. Kosasih , M. Karyana , A. Neal , and C. Y. Lau , “Review of Current COVID‐19 Diagnostics and Opportunities for Further Development,” Frontiers in Medicine 8, (2021), https://www.frontiersin.org/articles/10.3389/fmed.2021.615099. 10.3389/fmed.2021.615099 PMC8138031 34026773

[5] R. W. Peeling , C. J. Wedderburn , P. J. Garcia , et al., “Serology Testing in the COVID‐19 Pandemic Response,” Lancet Infectious Diseases 20, no. 9 (2020): e245–e249. 32687805 10.1016/S1473-3099(20)30517-X PMC7367660

[6] T. Fox , J. Geppert , J. Dinnes , et al. Antibody tests for identification of current and past infection with SARS‐CoV‐2 ‐ Fox, T ‐ 2022 | Cochrane Library, [cited 2024 Oct 21], https://www.cochranelibrary.com/cdsr/doi/10.1002/14651858.CD013652.pub2/full. 10.1002/14651858.CD013652.pub2 PMC9671206 36394900

[7] T. W. Heyming , D. Nugent , A. Tongol , et al., “Rapid Antibody Testing for SARS‐CoV‐2 Vaccine Response in Pediatric Healthcare Workers,” International Journal of Infectious Diseases 113 (2021): 1–6. 34601142 10.1016/j.ijid.2021.09.065 PMC8482557

[8] M. Churiwal , K. D. Lin , S. Khan , et al., “Assessment of the Field Utility of a Rapid Point‐of‐Care Test for SARS‐CoV‐2 Antibodies in a Household Cohort,” American Journal of Tropical Medicine and Hygiene 106, no. 1 (2022): 156–159. 10.4269/ajtmh.21-0592 PMC8733539 34818625

[9] Z. Li , Y. Yi , X. Luo , et al., “Development and Clinical Application of a Rapid IgM‐IgG Combined Antibody Test for SARS‐CoV‐2 Infection Diagnosis,” Journal of Medical Virology 92, no. 9 (2020): 1518–1524. 32104917 10.1002/jmv.25727 PMC7228300

[10] G. S. Ribeiro , M. Kikuti , L. B. Tauro , et al., “Does Immunity After Zika Virus Infection Cross‐Protect Against Dengue?,” Lancet Global Health 6, no. 2 (2018): e140–e141. 29389533 10.1016/S2214-109X(17)30496-5 PMC13032803

[11] M. M. O. Silva , L. B. Tauro , M. Kikuti , et al., “Concomitant Transmission of Dengue, Chikungunya, and Zika Viruses in Brazil: Clinical and Epidemiological Findings From Surveillance for Acute Febrile Illness,” Clinical Infectious Diseases 69, no. 8 (2018): 1353–1359. 10.1093/cid/ciy1083 PMC7348233 30561554

[12] L. C. Jacob‐Nascimento , M. M. Portilho , R. O. Anjos , et al., “Detection of Chikungunya Virus RNA in Oral Fluid and Urine: An Alternative Approach to Diagnosis?,” Viruses 16, no. 2 (2024): 235. 38400011 10.3390/v16020235 PMC10891727

[13] G. Cota , M. L. Freire , C. S. de Souza , et al., “Diagnostic Performance of Commercially Available COVID‐19 Serology Tests in Brazil,” International Journal of Infectious Diseases 101 (2020): 382–390. 33039612 10.1016/j.ijid.2020.10.008 PMC7544564

[14] W. P. O. S. Bernardes , T. G. Santos , N. M. G. S. Fernandes , et al., “Comparison of Diagnostic Performance of RT‐qPCR, RT‐LAMP and IgM/IgG Rapid Tests for Detection of SARS‐CoV‐2 Among Healthcare Workers in Brazil,” Journal of Infection and Public Health 16, no. 7 (2023): 1081–1088. 37210925 10.1016/j.jiph.2023.05.009 PMC10170900

[15] R. Batra , L. G. Olivieri , D. Rubin , et al., “A Comparative Evaluation Between the Abbott Panbio^TM^ COVID‐19 IgG/IgM Rapid Test Device and Abbott Architect^TM^ SARS CoV‐2 IgG Assay,” Journal of Clinical Virology 132 (2020): 104645. 32961429 10.1016/j.jcv.2020.104645 PMC7493757

[16] H. Haguet , J. Douxfils , C. Eucher , et al., “Clinical Performance of the Panbio Assay for the Detection of SARS‐CoV‐2 IgM and IgG in COVID‐19 Patients,” Journal of Medical Virology 93, no. 5 (2021): 3277–3281. 33599299 10.1002/jmv.26884 PMC8014867

[17] P. Escribano , A. Álvarez‐Uría , R. Alonso , et al., “Detection of SARS‐CoV‐2 Antibodies is Insufficient for the Diagnosis of Active or Cured COVID‐19,” Scientific Reports 10, no. 1 (2020): 19893. 33199713 10.1038/s41598-020-76914-5 PMC7669901

[18] A. P. M. Franco‐Luiz , N. M. G. S. Fernandes , S. Silva TB de , et al., “Longitudinal Study of Humoral Immunity Against SARS‐CoV‐2 of Health Professionals in Brazil: The Impact of Booster Dose and Reinfection on Antibody Dynamics,” Frontiers in Immunology 14 (2023): 1220600. https://www.frontiersin.org/journals/immunology/articles/10.3389/fimmu.2023.1220600/full. 37520570 10.3389/fimmu.2023.1220600 PMC10376701

[19] Q. Gao , L. Bao , H. Mao , et al., “Development of an Inactivated Vaccine Candidate for SARS‐CoV‐2,” Science 369, no. 6499 (2020): 77–81. 32376603 10.1126/science.abc1932 PMC7202686

[20] P. M. Folegatti , K. J. Ewer , P. K. Aley , et al., “Safety and Immunogenicity of the ChAdOx1 nCoV‐19 Vaccine Against SARS‐CoV‐2: A Preliminary Report of a Phase 1/2, Single‐Blind, Randomised Controlled Trial,” Lancet 396, no. 10249 (2020): 467–478. 32702298 10.1016/S0140-6736(20)31604-4 PMC7445431

[21] F. P. Polack , S. J. Thomas , N. Kitchin , et al., “Safety and Efficacy of the BNT162b2 mRNA Covid‐19 Vaccine,” New England Journal of Medicine 383, no. 27 (2020): 2603–2615. 33301246 10.1056/NEJMoa2034577 PMC7745181

