# Supplementary material for: Performance of Three Commercial Rapid Diagnostic Tests for Detection of IgM and IgG Antibodies Against SARS‐CoV‐2
Source: Immun Inflamm Dis. 2026 Apr 26;14(4):e70441. doi: 10.1002/iid3.70441 (PMC13111801; doi:10.1002/iid3.70441)
Supplement: Supplementary file 1 — Supporting File 1 [file IID3-14-e70441-s001.docx]

| **Accuracy by group** | | **PANBIO™ COVID-19 IgG/IgM Rapid Test Device** | | | **Bio-Manguinhos TR COVID-19**  **(IgM-IgG)** | | | **Bio-Manguinhos TR DPP® COVID-19 IgM/IgG** | | |
| --- | --- | --- | --- | --- | --- | --- | --- | --- | --- | --- |
| **Sensitivity** | | **IgM** | **IgG** | **IgM or IgG** | **IgM** | **IgG** | **IgM or IgG** | **IgM** | **IgG** | **IgM or IgG** |
|  | | **Sensitivity in % (95% confidence interval)** | | | | | | | | |
| RT-PCR-confirmed SARS-CoV-2 infection, overall | | 6.6 (3.9-10.4) | 56.0 (49.7-62.2) | 57.6 (51.3-63.7) | 51.0 (44.7-57.2) | 53.3 (47.0-59.5) | 54.5 (48.2-60.7) | 23.7 (18.7-29.4) | 47.5 (41.2-53.8) | 51.8 (45.5-58.0) |
|  | Symptomatic, unvaccinated | 7.1 (3.9-11.6) | 56.6 (49.4-63.6) | 58.1 (50.9-65.0) | 51.0 (43.8-58.2) | 53.5 (46.3-60.6) | 54.6 (47.3-61.6) | 22.2 (16.6-28.7) | 47.0 (39.9-54.2) | 51.5 (44.3-58.7) |
|  | Symptomatic, vaccinated^1^ | 5.6 (0.7-18.7) | 75.0 (57.8-87.9) | 75.0 (57.8-87.9) | 75.0 (57.8-87.9) | 75.0 (57.8-87.9) | 75.0 (57.8-87.9) | 38.9 (23.1-56.5) | 63.9 (46.2-79.2) | 63.9 (46.2-79.2) |
|  | Asymptomatic | 4.3 (0.1-21.9) | 21.7 (7.5-43.7) | 26.1 (10.2-48.4) | 13.0 (2.8-33.6) | 17.4 (5.0-38.8) | 21.7 (7.5-43.7) | 13.0 (2.8-33.6) | 26.1 (10.2-48.4) | 34.8 (16.4-57.3) |
| **Specificity** | | **IgM** | **IgG** | **IgM and IgG** | **IgM** | **IgG** | **IgM and IgG** | **IgM** | **IgG** | **IgM and IgG** |
|  | | **Specificity in % (95% confidence interval)** | | | | | | | | |
| Non-SARS-CoV-2-infected or vaccinated individuals, overall | | 97.0 (93.6-98.9) | 99.0 (96.4-99.9) | 96.5 (92.9-98.6) | 98.0 (94.9-99.4) | 100 (98.2-100) | 98.0 (94.9-99.4) | 83.9 (78.1-88.7) | 95.5 (91.6-97.9) | 79.9 (73.7-85.2) |
|  | Convalescent ILI cases | 98.3 (90.8-100) | 100 (93.8-100) | 98.3 (90.8-100) | 98.3 (90.8-100) | 100 (93.8-100) | 98.3 (90.8-100) | 87.9 (76.7-95.0) | 98.3 (90.8-100) | 86.2 (74.6-93.9) |
|  | Convalescent arbovirus illness cases^2^ | 96.6 (88.1-99.6) | 98.3 (90.8-100) | 94.8 (85.6-98.9) | 96.6 (88.1-99.6) | 100 (93.8-100) | 96.6 (88.1-99.6) | 72.4 (59.1-83.3) | 94.8 (85.6-98.9) | 69.0 (55.5-80.5) |
|  | Healthy controls |  |  |  |  |  |  |  |  |  |
|  | Before the pandemic | 94.8 (85.6-98.9) | 98.3 (90.8-100) | 94.8 (85.6-98.9) | 98.3 (90.8-100) | 100 (93.8-100) | 98.3 (90.8-100) | 84.5 (72.6-92.7) | 93.1 (83.3-98.1) | 77.6 (64.7-87.5) |
|  | During the pandemic, unvaccinated^3^ | 100 (86.3-100) | 100 (86.3-100) | 100 (86.3-100) | 100 (86.3-100) | 100 (86.3-100) | 100 (86.3-100) | 100 (86.3-100) | 96.0 (79.6-99.9) | 96.0 (79.6-99.9) |

**Supplementary table 1.** Sensitivity, specificity and respective 95% confidence intervals of three commercially available rapid diagnostic tests for detection of IgM and IgG antibodies against SARS-CoV-2.

ILI = influenza-like illness

^1^ The vaccinated RT-PCR-confirmed COVID-19 cases have used at least one dose of the COVID-19 vaccine produced by AstraZeneca/Oxford or CoronaVac/Butantan.

^2^ The convalescent arbovirus illness cases include 29 RT-PCR-confirmed dengue cases and 29 RT-PCR-confirmed chikungunya cases, for which serum samples were obtained during convalescence (11-50 days post-onset of fever) in the pre-pandemic period.

^3^ All the non-vaccinated healthy controls included after the start of the COVID-19 pandemic denied having a diagnosis of COVID-19.
