# Supplementary material for: Performance of Three Commercial Rapid Diagnostic Tests for Detection of IgM and IgG Antibodies Against SARS‐CoV‐2
Source: Immun Inflamm Dis. 2026 Apr 26;14(4):e70441. doi: 10.1002/iid3.70441 (PMC13111801; doi:10.1002/iid3.70441)
Supplement: Supplementary file 2 — Supporting File 2 [file IID3-14-e70441-s003.docx]

**Supplementary Table 2.** Positivity rates and corresponding 95% confidence intervals for IgM and IgG antibody detection among COVID-19 vaccinated individuals using three commercial rapid diagnostic tests for SARS-CoV-2

| **Detection of antibodies among vaccinated healthy individuals ^1^** | **PANBIO™ COVID-19 IgG/IgM Rapid Test Device** | | | **Bio-Manguinhos TR COVID-19 (IgM-IgG)** | | | **Bio-Manguinhos TR DPP® COVID-19 IgM/IgG** | | |
| --- | --- | --- | --- | --- | --- | --- | --- | --- | --- |
| **Group** | **IgM** | **IgG** | **IgM or IgG** | **IgM** | **IgG** | **IgM or IgG** | **IgM** | **IgG** | **IgM or IgG** |
|  | **positivity rate in % (95% confidence interval)** | | | | | | | | |
| Overall | 5.3 (1.5-13.1) | 44.0 (32.5-5.9) | 48.0 (36.3-59.8) | 52.0 (40.2-63.7) | 48.0 (36.3-59.8) | 54.7 (42.7-66.2) | 12.0 (2.5-31.2) | 29.3 (19.4-41.0) | 38.7 (27.6-50.6) |
| By vaccine type |  |  |  |  |  |  |  |  |  |
| CoronaVac | 4.2 (0.1-21.1) | 66.7 (44.7-84.4) | 66.7 (44.7-84.4) | 58.3 (36.6-77.9) | 62.5 (40.6-81.2) | 62.5 (40.6-81.2) | 8.3 (1.0-27.0) | 37.5 (18.8-59.4) | 41.7 (22.1-63.4) |
| AstraZeneca/Oxford | 3.8 (0.1-19.6) | 34.6 (17.2-55.7) | 38.5 (20.2-59.4) | 50.0 (29.9-70.1) | 46.2 (26.6-66.6) | 53.8 (33.4-73.4) | 15.4 (4.4-34.9) | 30.8 (14.3-51.8) | 42.3 (23.4-63.1) |
| Pfizer (BioNTech) | 8.0 (1.0-26.0) | 32.0 (14.9-53.5) | 40.0 (21.1-61.3) | 48.0 (27.8-68.7) | 36.0 (18.0-57.5) | 48.0 (27.8-68.7) | 12.0 (2.5-31.2) | 20.0 (6.8-40.7) | 32.0 (14.9-53.5) |
| By the number of vaccine doses |  |  |  |  |  |  |  |  |  |
| 1 dose | 2.9 (0.1-15.3) | 44.1 (27.2-2.1) | 47.1 (29.8-64.9) | 52.9 (35.1-70.2) | 50.0 (32.4-67.6) | 55.9 (37.9-72.8) | 8.8 (1.9-23.7) | 32.4 (17.4-50.5) | 38.2 (22.2-56.4) |
| 2 doses | 7.3 (1.5-19.9) | 43.9 (28.5-60.3) | 48.8 (32.9-64.9) | 51.2 (35.1-67.1) | 46.3 (30.7-62.6) | 53.7 (37.4-69.3) | 14.6 (5.6-29.2) | 26.8 (14.2-42.9) | 39.0 (24.2-55.5) |
| By time since last vaccine dose ^2,3^ |  |  |  |  |  |  |  |  |  |
| <30 days | 5.7 (0.7-19.2) | 40.0 (23.9-57.9) | 45.7 (28.8-63.4) | 48.6 (31.4-66.0) | 42.9 (26.3-60.6) | 51.4 (34.0-68.6) | 11.4 (3.2-26.7) | 25.7 (12.5-43.3) | 37.1 (21.5-55.1) |
| 30 - 89 days | 12.5 (1.6-38.3) | 37.5 (15.2-64.6) | 43.8 (19.8-70.1) | 50.0 (24.7-75.3) | 43.8 (19.8-70.1) | 50.0 (24.7-75.3) | 12.5 (1.6-38.3) | 25.0 (7.3-52.4) | 31.3 (11.0-58.7) |
| ≥90 days | 0.0 (0.0-14.8) | 52.2 (30.6-73.2) | 52.2 (30.6-73.2) | 56.5 (34.5-76.8) | 56.5 (34.5-76.8) | 60.9 (38.5-80.3) | 8.7 (1.1-28.0) | 39.1 (19.1-61.5) | 43.5 (23.2-65.5) |

^1^ Vaccinated healthy individuals were enrolled after the start of the COVID-19 pandemic, reported no prior diagnosis of COVID-19, and had received at least one dose of one of the following vaccines: CoronaVac (Sinovac/Butantan), AstraZeneca/Oxford (Fiocruz), or Pfizer/BioNTech.

^2^ Time between the last vaccine dose and sample collection.

^3^ It was not possible to retrieve the vaccination date for one patient.
